# Microstructural Changes in Patients With Parkinson's Disease Comorbid With REM Sleep Behaviour Disorder and Depressive Symptoms

**DOI:** 10.3389/fneur.2018.00441

**Published:** 2018-06-26

**Authors:** Farzaneh Ghazi Sherbaf, Yasna Rostam Abadi, Mahtab Mojtahed Zadeh, Amir Ashraf-Ganjouei, Hossein Sanjari Moghaddam, Mohammad Hadi Aarabi

**Affiliations:** Faculty of Medicine, Tehran University of Medical Sciences, Tehran, Iran

**Keywords:** Parkinson's disease, REM sleep behavior disorder, depression, connectometry, diffusion MRI

## Abstract

The diagnosis of Parkinson's disease (PD) is currently anchored on clinical motor symptoms, which appear more than 20 years after initiation of the neurotoxicity. Extra-nigral involvement in the onset of PD with probable nonmotor manifestations before the development of motor signs, lead us to the preclinical (asymptomatic) or prodromal stages of the disease (various nonmotor or subtle motor signs). REM sleep behavior disorder (RBD) and depression are established prodromal clinical markers of PD and predict worse motor and cognitive outcomes. Nevertheless, taken by themselves, these markers are not yet claimed to be practical in identifying high-risk individuals. Combining promising markers may be helpful in a reliable diagnosis of early PD. Therefore, we aimed to detect neural correlates of RBD and depression in 93 treatment-naïve and non-demented early PD by means of diffusion MRI connectometry. Comparing four groups of PD patients with or without comorbid RBD and/or depressive symptoms with each other and with 31 healthy controls, we found that these two non-motor symptoms are associated with lower connectivity in several white matter tracts including the cerebellar peduncles, corpus callosum and long association fibers such as cingulum, fornix, and inferior longitudinal fasciculus. For the first time, we were able to detect the involvement of short association fibers (U-fibers) in PD neurodegenerative process. Longitudinal studies on larger sample groups are needed to further investigate the reported associations.

## Introduction

Parkinson's disease (PD), a form of α-synucleinopathy neurodegeneration (1), is manifested by a heterogeneous combination of motor and non-motor symptoms (NMS) (2). PD is classically diagnosed based on its cardinal motor symptoms relatively late in the course of the disease, years or even decades after the initiation of neurotoxicity. Thus, the golden time to halt the disease progression is missed. Searching for markers to diagnose PD in the early stage of the disease, a critical opportunity for neuroprotective interventions remains a hot topic (3).

Rapid Eye movement (REM) sleep behavior disorder (RBD), characterized by unpleasant dreams and loss of normal muscle atonia (4), has by far proved to be the strongest precursor of upcoming PD (3) and with more than 75% conversion rate, is considered as an evolving synucleinopathy (5–7). With an estimated prevalence of 15–60% in PD patients (8), baseline RBD is attributed to more aggressive clinical subtype with worse motor and non-motor symptoms, especially depressive disorders and cognitive decline (9). Besides high specificity and prognostic value, its low predictive sensitivity and long lead time to the development of parkinsonism bring challenges in practice. Combining RBD with another prodromal symptom may solve this task by increasing the risk of conversion (10).

Depression is another established clinical prodromal marker (3, 7) and is the main culprit in a lower quality of life in PD patients (11). Depression together with RBD play as potential interactive risk factors for the development of dementia in PD which is associated with more advanced disease and poorer prognosis (12–14). Depression has multitude neural and clinical correlates with RBD. Depressed mood is often associated with disrupted REM sleep structure such as decreased latency, longer duration and more rapid eye movements, which may precede the onset of depressive episodes or even persist after complete remission with an increased rate of relapse or recurrence and poor treatment response (15–18). Furthermore, studies on healthy relatives of depressed patients have shown that REM sleep disturbances can predict the development of depressive episodes (19–22). This indicates that REM sleep dysregulation is not merely secondary but rather share underlying pathologies with depression (23–25). The frequency of Depression and its severity are also shown to be related to RBD and other sleep disturbances in PD (9, 26, 27). Some studies on depressed patients with idiopathic RBD (iRBD) have supported the assumption that this comorbidity might underpin and accelerate the neurodegenerative process (28–30). Interestingly, Wing et al. proved depression as a potential predictor of upcoming PD in following a cohort of iRBD patients (31). Remarked subcortical Lewy bodies in late-life depression (32) further supports this proposed link between depression and RBD.

Accumulating evidence suggests that white matter damage underlies the heterogenous manifestation of PD symptomatology (33). Although the pathogenesis of PD is still unclear, it is speculated that α-synuclein species spread as a prion-like pattern through axons and cause disruption in the white matter integrity via mitochondrial damage and glial activation (34). In this regard, diffusion MRI (dMRI) is a promising tool to measure white matter microstructure *in vivo* and has shed light through the knowledge of involved neural networks in PD in association with its distinct features. DMRI connectomery is a powerful analytical method that probes significant between-group differences within subcomponents of a neural pathway, rather than the entire pathway. Conventional diffusion tensor approaches track the entire pathway, which will inevitably contain fibers not strongly associated with study variables. This will result in higher sensitivity and lower type II error using connectometry approach by focusing only on significant variabilities (35). Furthermore, connectometry relies on Spin Distribution Function (SDF) to measure the density of water diffusion for any direction of a voxel and reveals the so-called “local connectome fingerprint” which is highly specific to each individual (36). Ability to quantify the degree of connectivity between adjacent voxels within a neural fascicle, local connectome, has opened a new door to investigate pathological insults on the unique configuration of white matter microstructure.

In two previous studies, we have tried to discover whether RBD and depression can lead us to white matter degeneration signature of early PD, comparing two groups of depressed (dPD) and non-depressed PD patients (ndPD) with comorbid RBD (37) and comparing two groups of dPD and ndPD without comorbid RBD (38) through dMRI connectometry. RBD and depressive symptoms both have been proposed as markers of prodromal PD and with possible cumulative effect on progression to PD and its severe subtypes and each can predict worse outcomes in PD or the other. In this study, which is an extension of two previous mentioned studies, we aimed to track differences in white matter connectivity in four groups of treatment-naïve early PD patients with and without comorbid RBD and/or depressive symptoms compared to healthy controls (HC) with added within PD subgroups comparisons.

## Materials and methods

### Participants

Participants, PD patients and HC, involved in this research were recruited from Parkinson's Progression Markers Initiative (PPMI, http://www.ppmi-info.org/). The study was approved by the institutional review board of all participating sites. Written informed consent was obtained from all participants before study enrolment. The study was performed in accordance with relevant guidelines and regulations (39). These participants were tested and confirmed negative for any neurological disorders apart from PD. The participants' PD status was confirmed by Movement Disorder Society-Unified Parkinson's Disease Rating Scale (MDS-UPDRS), and the loss of dopaminergic neurons was observed on DAT scans.

We analyzed only drug-naïve cases with available diffusion weighted imaging (DWI) in baseline visit after performing automated quality-control steps expressed by Fang-Chen Yeh, using q-space diffeomorphic reconstruction (QSDR) (40). This method is based on checking how compatible the quantitative anisotropy (QA) value of each voxel is with the reconstructed QA map. Subjects were excluded if imaging failed specific quality control criteria. Finally, a total of 93 drug-naïve early PD patients and 31 age-matched and sex-matched HC with good imaging quality were enrolled in this study. Clinical measures included disease duration, motor section (III) of UPDRS, Hoehn and Yahr (H&Y staging), Montreal Cognitive Assessment (MoCA), Epworth Sleepiness Scale (ESS) for daytime sleepiness, and the University of Pennsylvania Smell Identification Test (UPSIT) for olfaction function. Depression was assessed using the Geriatric Depression Scale (GDS), with a cut-off score of 5 or more indicating clinically significant symptoms (41). GDS is an easy to use, self-report screening and diagnostic tool with good reliability and validity to discriminate minor and major depressive disorders from non-depressive disorder in PD patients of all ages, particularly elders (41). It is recommended for use by the Movement Disorders Society to screen for symptoms of depression in PD individuals (www.movementdisorders.org/MDS/Education/Rating-Scales.htm). RBD was assessed using REM Sleep Behavior Disorder Screening Questionnaire (RBDSQ), with a cut-off score of 5 or above to detect probable RBD (42, 43). Based on GDS and RBDSQ, PD patients were divided into four groups of 14 patients with depression and RBD (DEP+/RBD+), 16 without depression and with RBD (DEP-/RBD+), 43 with depression and without RBD (DEP+/RBD-), and 20 without depression and without RBD (DEP-/RBD-).

### Data acquisition

Data used in the preparation of this article were obtained from PPMI database (www.ppmiinfo.org/data) (39). This dataset was acquired on 3 Tesla Siemens scanners, producing 64 diffusion MRI (repetition time = 7748 MS, echo time = 86 ms; voxel size: 2.0 × 2.0 × 2.0 mm3; field of view = 224 × 224 mm) at b = 1,000 s/mm^2^ and one b0 image along with 3D T1-weighted structural scans (repetition time = 8.2 ms, echo time = 3.7 ms; flip angle = 8°, voxel size: 1.0 × 1.0 × 1.0 mm^3^; field of view = 240 mm, acquisition matrix = 240 × 240).

### Diffusion MRI processing

The diffusion MRI data were corrected for subject motion, eddy current distortions, and susceptibility artifacts due to the magnetic field inhomogeneity using Explore DTI toolbox (44). We performed quality control analysis on the subject's signals based on the goodness-of-fit value given in QSDR reconstruction of fibers. Each QSDR reconstruction file has a goodness-of-fit value quantified by R2. For example, an R82 indicates a goodness-of-fit between of the subject and template of 0.82 total. We excluded cases in which the R2 value did not reach a threshold of 0.6 otherwise.

### Diffusion MRI connectometry

The diffusion data were reconstructed in the Montreal Neurological Institute (MNI) space using QSDR to obtain the SDF (45), to detect the differences between groups. Quantitative anisotropy (QA) is one of the several diffusion indices derived from spin density, i.e., SDF (46). QA of each fiber orientation gives the peak value of water density in that direction. More precisely, in contrast to tensor-derived measures such as fractional anisotropy (FA) which are defined for each voxel and rely on diffusivity, QA is defined for each fiber orientation and is based on density. Therefore, diffusivity measures reflect the intactness of fibers, while QA quantifies the total diffusing water or “connectivity.” As a result, QA has successfully overcome the shortage of conventional tensor measures on crossing fibers. Another advantage of QA over diffusivity metrics is that it is not affected by partial volume defect, as it is derived from spin density (47). We used diffusion MRI connectometry to identify white matter tracts in which QA was significantly different between two groups of PD patients with different degrees of depression and RBD, and comparing each PD group to HC. Resulting uncorrected output was corrected for multiple comparisons by false discovery rate (FDR). A deterministic fiber tracking algorithm was conducted along the core pathway of the fiber bundle to connect the selected local connectomes (48). Tracts with QA > 0.1, angle threshold lesser than 40° and tract length >40 mm were included. To estimate the false discovery rate, a total of 2,000 randomized permutations were applied to the group label to obtain the null distribution of the track length. A T-score threshold of 2.5 was assigned to select local connectomes, and the local connectomes were tracked using a deterministic fiber tracking algorithm. Permutation testing allows for estimating and correcting the FDR of Type-I error inflation due to multiple comparisons. The analysis was conducted using publicly available software DSI Studio (http://dsi-studio.labsolver.org), released in 5th April 2018.

### Statistical analysis

IBM SPSS Statistics for Windows, version 22 (IBM Corp., Armonk, N.Y., USA) was used to analyse the demographic and clinical data. Probability graphics and Shapiro–Wilk test were used to check the compliance of variables with normal distribution. For normally distributed variables, one-way analysis of variance (ANOVA) was used to assess differences of means between groups. Kruskal–Wallis test was used to determine whether there are any statistically significant differences within continuous variables without normal distribution. Pearson's chi-square was used to test nominal variables across groups. Finally, *P* < 0.05 were considered statistically significant.

## Results

### Demographic and clinical measures

PD patients in four groups were matched in their age, sex, disease duration and years of education. Patients were also comparable based on their degree of motor impairment (UDPRS-III and H&Y) after controlling for age, sex, and disease duration. The cognitive state (MoCA), olfaction function (UPSIT), and daytime sleepiness (ESS) did not differ between four PD groups (Table 1). There are few discrepancies between PD patients enrolled in this study and those investigated in our two previous studies, as we attempted to include patients matched in their demographic, motor and other non-motor symptoms other than depression and RBD in the present study. Eleven patients in the DEP+/RBD+ group, eight patients in the DEP-/RBD+ group, 19 patients in the DEP+/RBD- group, and 14 patients in the DEP-/RBD- group were at their stage 2 of H&Y, indicative of bilateral involvement without disturbance in balance. The rest of the patients were all in stage 1 of H&Y scaling compatible with mild symptoms of unilateral involvement. None of the patients in any group were demented as they all scored above the cut-off score of 21 on the MoCA. HC were matched with PD patients regarding age, sex, handedness, education years, MoCA, and ESS scores, while performed better than PD patients on GDS, RBD, and UPSIT.

**Table 1:** Demographic and baseline clinical information of healthy controls and patients with Parkinson's disease with or without comorbid RBD and/or depression.

**Groups**	**Healthy controls (*n* = 31)**	**DEP+RBD+ (*n* = 14)**	**DEP-RBD+ (*n* = 16)**	**DEP+RBD- (*n* = 43)**	**DEP-RBD- (*n* = 20)**	*p*-Value^**^	*p*-Value (between PD groups)^**^
Age (mean ± *sd*)	58.0 ± 12.1	58.8 ± 9.8	59.2 ± 11.6	58.5 ± 8.7	58.4 ± 9.4	0.997	0.997
Female/Male no.	18/13	11/3	12/4	24/19	13/7	0.441	0.441
Handedness (L/R)	3/28	0/14	2/14	4/36	3/17	0.717	0.716
Education years (mean ± *sd*)	15.0 ± 2.8	15.1 ± 2.4	16.2 ± 2.6	15.0 ± 3.0	14.8.±3.1	0.611	0.611
Duration of disease in years (mean ± *sd*)	–	7.5 ± 7.5	8.5 ± 7.5	6.2 ± 6.7	6.3 ± 6.8	–	0.738
Hoehn & Yahr stage (mean ± *sd*)	–	1.8 ± 0.4	1.5 ± 0.5	1.4 ± 0.5	1.7 ± 0.5	–	0.146
UPDRS III^*^ (mean ± *sd*)	–	21.7 ± 8.3	21.8 ± 11.4	19.3 ± 7.3	24.7±.8.7	–	0.163
MOCA^*^ score (mean ± *sd*)	28.4 ± 1.1	27.4 ± 2.2	27.5 ± 1.8	27.6 ± 1.8	27.6 ± 2.4	0.324	0.955
RBD^*^ score (mean ± *sd*)	3.4 ± 2.1	7.4 ± 2.0	7.1 ± 1.4	2.5 ± 1.1	2.3 ± 1.1	<**0.001**	<**0.001**
GDS^*^ score (mean ± *sd*)	4.4 ± 1.1	5.1 ± 0.9	3.2 ± 1.1	5.2 ± 0.4	3.3 ± 1.1	<**0.001**	<**0.001**
ESS^*^ scale (mean ± *sd*)	6.7 ± 4.3	7.4 ± 3.1	7.5 ± 3.8	5.5 ± 3.2	6.2 ± 3.0	0.263	0.126
UPSIT (mean ± *sd*)	33.1 ± 4.3	21.6 ± 9.9	19.6 ± 8.4	24.8 ± 8.1	24.3 ± 6.8	<**0.001**	0.145

* UPDRS III, Unified Parkinson's Disease Rating Scale part III; ESS, Epworth Sleepiness Scale; MoCA, Montreal Cognitive Assessment; RBD, REM sleep Behaviour Disorder Screening Questionnaire; GDS, Geriatric Depression Scale; UPSIT, the University of Pennsylvania Smell Identification Test (UPSIT).

** *p-value of one-way ANOVA analysis for age, education years, disease duration, and UPSIT; Pearson Chi-square for gender, handedness, and H&Y stage; and Kruskal–Wallis test for ESS, UPRDS part III, GDS scale, MoCA score, and RBDSQ. P < 0.05 are considered statistically significant*.

### PD groups vs. HC imaging analysis

As outlined in Table 2, all four groups of PD patients showed lower connectivity in superior longitudinal fasciculus and U-fibers of parietal lobe and motor and pre-motor areas of the frontal lobe. Left inferior longitudinal fasciculus (ILF) was only disrupted in the PD groups with comorbid depression, i.e., DEP+/RBD- and DEP+/RBD+. Cingulum had lower connectivity in all PD patients except RBD+/DEP+. This group instead showed lower connectivity in the body of corpus callosum (CC).

**Table 2:** Regions with significantly reduced quantitative anisotropy comparing each group of PD patients with healthy controls.

**PD RBD+/DEP+ vs. HC (FDR = 0.01)**	**PD Dep–/RBD+ vs. HC (FDR = 0.02)**	**Dep+/RBD–vs. HC (FDR = 0.001)**	**DEP-/RBD-vs. HC (FDR = 0.036)**
B-SLF	B-SLF	B-SLF	B-SLF
B-U-fiber	B-U-fiber	L-U-fiber	R-U-fiber
L-ILF	B-cingulum	L-ILF	R-cingulum
body of CC		B-cingulum	

*B, bilateral; L, left; R, right; SLF, superior longitudinal fasciculus; ILF, inferior longitudinal fasciculus; CC, corpus callosum; FDR, false discovery rate*.

### Between-group imaging analyses of PD patients

**Figure 1:**
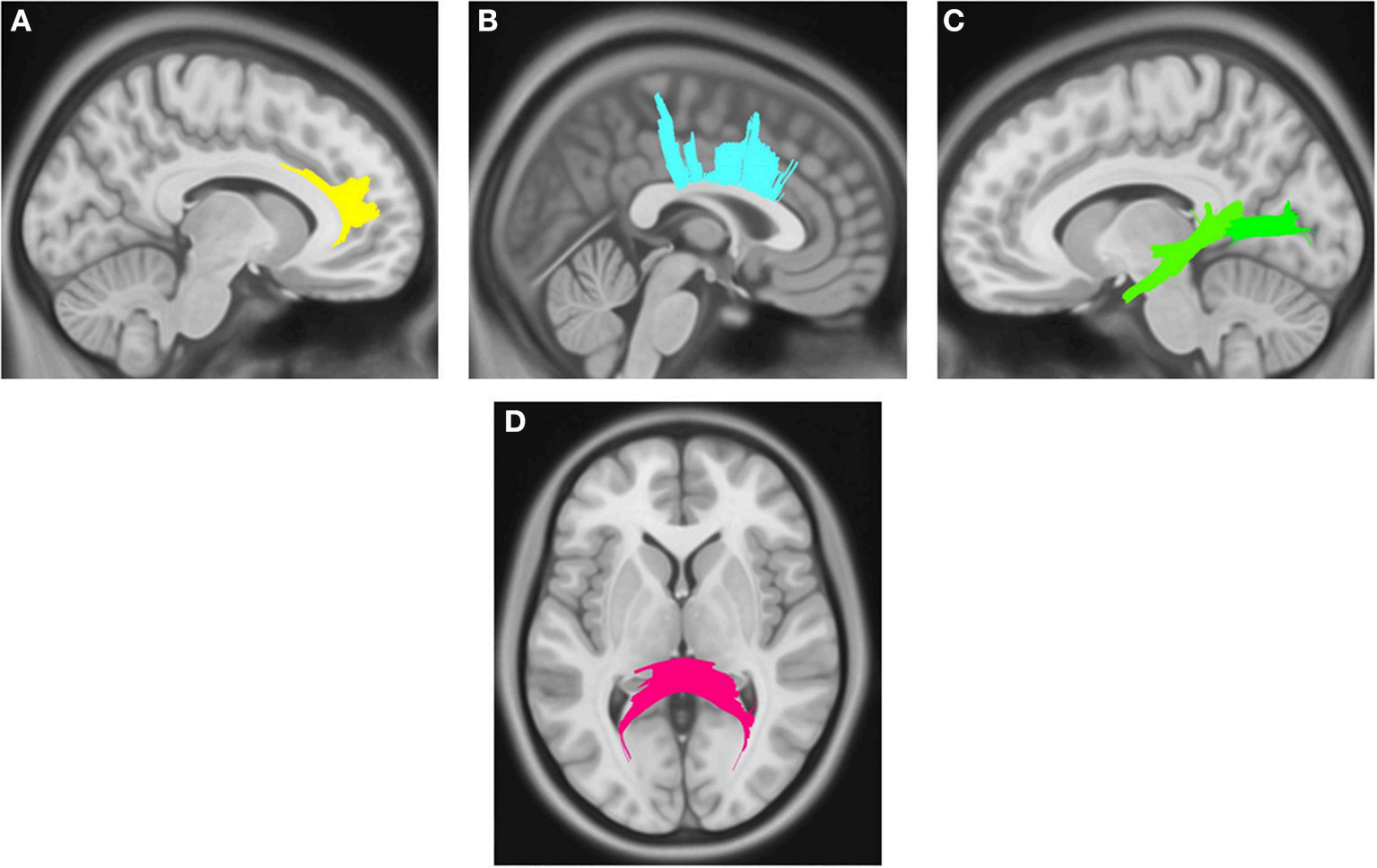
White matter pathways with significantly reduced quantitative anisotropy in PD DEP+RBD+ vs. PD DEP-RBD+ (FDR = 0.03). **(A)** right cingulum, **(B)** body of the corpus callosum, **(C)** left inferior longitudinal fasciculus, **(D)** splenium. The results are overlaid on ICBM152 (mni_icbm152_t1) from the McConnell Brain Imaging Centre using DSI-STUDIO software.

**Figure 2:**
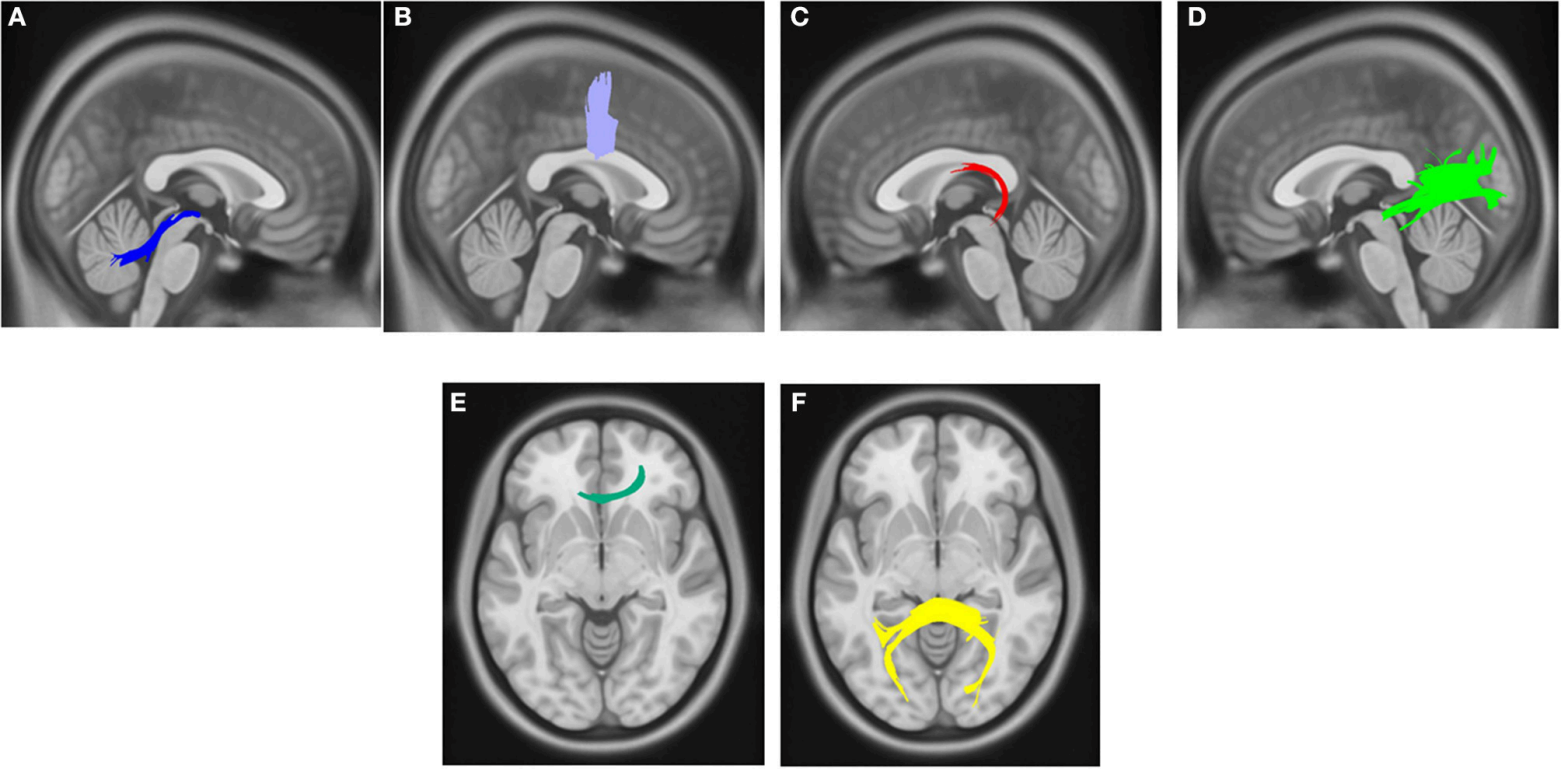
White matter pathways with significantly reduced quantitative anisotropy in PD DEP+RBD+ vs. PD DEP+RBD- (FDR = 0.02). **(A)** right superior cerebellar peduncle, **(B)** body of the corpus callosum, **(C)** left fornix, **(D)** left inferior longitudinal fasciculus, **(E)** genu, **(F)** splenium. The results are overlaid on ICBM152 (mni_icbm152_t1) from the McConnell Brain Imaging Centre using DSI-STUDIO software.

Compared with PD DEP-/RBD+ patients, PD DEP+/RBD+ patients showed decreased connectivity in the right cingulum, left ILF, splenium, and body of the CC (FDR = 0.03) (Figure 1). As shown in Figure 2, the PD DEP+/RBD+ group demonstrated decreased connectivity in the genu, splenium, and body of CC, left ILF, left fornix, and right superior cerebellar peduncle (SCP) in contrast to PD DEP+/RBD- (FDR = 0.02). The group differences between PD DEP+/RBD+ patients and PD DEP-/RBD- were that connectivity in PD DEP-/RBD- was higher than that in PD DEP+/RBD+ patients in the genu, splenium and body of CC, bilateral cingulum, left ILF, left fornix, right SCP, and right inferior fronto-occipital fasciculus (IFOF) (FDR = 0.01).

Compared with PD DEP-/RBD- patients, PD DEP–/RBD+ patients showed decreased connectivity in the bilateral cingulum, bilateral fornix, left ILF, genu, and body of CC, middle cerebellar peduncle (MCP), bilateral SCP, right uncinate fasciculus (UF), and left cerebro-cortical pathway (CST) (FDR = 0.03). These were almost the same results of comparison between PD DEP+/RBD- with the DEP-/RBD+, except for the right fornix, left cingulum, and left ILF (FDR = 0.006).

Finally, PD DEP+/RBD- patients showed decreased connectivity in the bilateral cingulum, bilateral fornix, bilateral ILF, left UF, right CST and genu, splenium, and body of CC compared to PD DEP-/RBD- (FDR = 0.02). Table 3 summarizes the significant regions of lower connectivity in between-group analyses.

**Table 3:** Regions with significantly different connectivity in between group comparing of PD patients with or without comorbid RBD and/or depression.

***PD DEP*+*/RBD*+ vs. *PD DEP-/RBD–* (FDR = 0.01)**	***PD DEP*+*/RBD*+ vs. *PD DEP–/RBD*+ (FDR = 0.03)**	***PD DEP*+*/RBD*+ vs. *PD DEP*+*/RBD–* (FDR = 0.02)**
R-SCP B-cingulum L-fornix L-ILF R-IFOF Genu, body and splenium of CC	R-Cingulum L-ILF Splenium and body of CC (previous study: Fornix, genu, L-MCP, R-CST, R-cing, L-ILF)	R-SCP L-ILF L-fornix Genu, body and splenium of CC
***PD DEP–/RBD***+ **vs**. ***PD DEP-/RBD–*** **(FDR** = **0.03)**	***PD DEP***+***/RBD–*** **vs. PD** ***DEP-/RBD-*** **(FDR** = **0.02)**	***PD DEP–/RBD***+ **vs**. ***PD DEP***+***/RBD–*** **(FDR** = **0.006)**
B-SCP MCP B-cingulum B-fornix L-ILF Genu and body of CC R-UF L-CST	B-cingulum B-fornix B-ILF Genu, body and splenium of CC L-UF R-CST (Previous study: R-IFOF, MCP Genu, UF, L-ILF, R-CST, Fornix)	B-SCP MCP R-cingulum L-fornix Genu and body of CC L-external capsule L-CST

*The left groups vs. the right groups showed lower quantitative anisotropy in the areas listed. B, bilateral; R, right; L, left; SCP, superior cerebellar peduncle; MCP, middle cerebellar peduncle; ILF, inferior longitudinal fasciculus; UF, uncinate fasciculus; CST, cerebrospinal tract; IFOF, inferior fronto-occipital fasciculus; CC, corpus callosum; FDR, false discovery rate*.

## Discussion

In this study, we investigated the neural underpinnings of RBD and depression as clinical PD prodromal markers with more severe outcome mainly of the motor and cognitive function. RBD not only predicts upcoming PD but also warns the development of non-tremor dominant motor subtype with a diversity of other NMS such as depression and dementia (49). Recent evidence has exposed the role of widespread white matter disruption underlying heterogenous symptoms of PD, including commissural, projection and long association fibers (33). Previous studies have mostly relied on diffusion tensor imaging (DTI), which has major limitations in detecting pathologies in areas of high crossing fibers such as near cortical structures. Using connectometry analysis, which successfully overcomes this pitfall (50), we were able to capture the novel contribution of U-fibers in PD. U-fibers are short association fibers which run between white matter and cortex and connect adjacent gyri and participate in higher functions of the brain. As these particular fibers have low metabolic rate and high blood supply, they are relatively spared in vascular disorders such as stroke. In contrast, pathologies with glial insult, such as multiple sclerosis, are shown to result in early involvement of U-fibers and the subsequent cognitive malperformance in such patients (51). Glial dysfunction is one of the key events in initiation and progression of neurodegenerative processes as well (52–54). Therefore, it seems that U-fibers can be potentially affected in PD. This is a thought-provoking result that should be addressed in future research.

Among other fibers with lower connectivity, like U-fibers, SLF also consistently differed between PD patients and HC regardless of the comorbid RBD or depression. Previous studies have specified the lower integrity of SLF associated with several domains of cognitive decline from mild cognitive impairment to dementia (55–58), and also in non-tremor motor phenotypes of PD such as freezing of gait (59, 60), bradykinesia (61), and postural instability (62), which have poor prognostic implications. SLF is a long association fiber that originates from posterior regions of the brain and projects to the frontal lobe. However, among few studies which have investigated DTI findings in relation to depression, lower FA in the left SLF near the midline and superior frontal lobe is reported in dPD versus ndPD in one whole brain study (63). The authors have discussed this finding through the cognitive aspect of depression. A meta-analysis has also implicated the disruption of SLF in major depressive disorder (MDD) (64). However, within-patients analysis of our cohort did not reveal any subgroup-differences in SLF, which would indirectly point toward to non-contribution of this tract with depression and RBD. This is in agreement with our previous studies on dPD (37, 38, 65).

ILF was only disrupted in the PD groups with comorbid depression compared to HC. ILF fast and directly integrates visual categorization and recognition data between extrastriate visual cortex and temporal gyri which subsequently project to the limbic structures (66). High-order visual problems such as visually evoked memory and emotional impairments are linked to ILF disruptions, as a key component of the visual-limbic pathway (67, 68). ILF is among the main fibers involved in the major depressive disorder, proved by the mentioned meta-analysis of whole brain voxel-based DTI studies (64). The same association was also demonstrated in dPD (63, 69) and mild cognitive impairment and dementia in PD (55, 58) based on whole brain tract-based spatial statistics studies. This finding may signal the higher risk of cognitive impairment in terms of executive and visuospatial dysfunction in dPD. ILF disruption most consistently observed in the left hemisphere is in agreement with the perception that depression is a result of left hemispheric dysfunction (70). Interestingly, it is revealed that right-onset PD predict more severe depressive symptoms in the course of the disease (71). These may explain our finding of ILF lateralization in comorbid depression in PD. Apart from ILF alterations in dPD, our results are also indicative of reduced connectivity in left ILF in DEP-/RBD+ compared to DEP-/RBD-, which is in line with the study by Ford et al. (72). However, few other studies comparing PD-RBD patients with PD-non-RBD have not reached to this association. May longitudinal follow-up of these patients reveal subsequent depressive symptoms followed by ILF disruption, should be investigated in well-designed cohorts.

Cingulate, the prominent limbic structure and the well-known structure of emotion and cognition activates during REM sleep (73). Cingulum injury has been shown in abnormalities in attention, memory and emotional processing (74). Existing literature is already enriched with cingulum associations in depression and its pathognomonic REM sleep dysregulations (75, 76) and also depression, apathy, impulse control deficit and dementia in PD (77–80). More severe lesions of this tract predispose to poorer treatment response in late-life depression (81) and is a stimulating target to treat resistant depression (82). This area is also attributed to comorbid RBD in PD patients recruited from PPMI database (83, 84). Overall, it is not surprising that disrupted cingulum bundle is correlated with depressive symptoms and RBD in PD patients.

CC with more than 200 million axonal projections, is the largest fiber bundle in the central nervous system, which actively transfers information between homologous areas of two cerebral hemispheres (85). This commissure has a major role in the regulation of cognitive and emotional function and bilateral limb movement (86). Extensive corpus callosal damage is described in early PD (87), which becomes more severe with motor worsening (88). Reduction of callosal integrity is implicated in the freezing of gait and postural instability in non-tremor dominant PD (89). This phenotype is more often accompanied by cognitive decline and mood disorders (33, 90). In line with these observations, diffusion MRI connectometry has revealed reduced integrity in CC in the neuropathology of comorbid RBD (84) and depression (65) in PD. Same association is shown regarding cognitive decline and its severity in PD (33).

The results of between PD subgroups evaluation showed that superior and middle cerebellar peduncles have lower connectivity consistently comparing PD patients with RBD compared to those without RBD. Cerebellum has heavy connections to the cerebral cortex via brainstem structures. Disruptions of this circuitry and asynchronization of cerebral and cerebellar functions are related to sleep-wake state abnormalities (91, 92). As a result, cerebellar pathology is often present in sleep disorders (93), and its cortical volume reduction has been shown in RBD (83, 94), although the exact contributed pathways are yet to be elucidated. It is now well documented that sleep has a major role in memory consolidation (95). Cerebellar increased activity during sleep in order to integrate learned motor skills is well-documented (96), and gray matter reductions of the cerebellum have resulted in the impaired consolidation of action memories (97). The interconnected sleep and cerebellar cognitive and motor-related functions may point toward the more severe motor and cognitive impairments associated with PD-RBD (9). Metabolic imaging studies have interestingly proposed cerebellum as a PD prodromal biomarker, as a part of a metabolic network associated with the severe motor subtype of PD (98). Idiopathic RBD patients with altered cerebellar metabolism are also at higher risk of photoconverting to neurodegeneration (99). Our consistent results of significant altered white matter in cerebellar peduncles is in line with disturbed cortico-cerebellar connections in PD-RBD patients in contrast to PD patients without RBD. Another DWI connectometry analysis also has manifested middle cerebellar peduncle as a discriminative indicator between these two groups of patients recruited from PPMI (84). Recent neuroanatomical studies have shown the important role of the cerebellum in emotional regulation and high-order cognitive coordination through extensive networks with the cerebral cortex, limbic system and thalamus via superior and middle cerebellar peduncles (100–105). There is a tendency to lateralization in the cerebellum in processing cognition and affection. Lesions of the right cerebellum, in connection to the left cerebral cortex, result in cognitive dysfunctions and positive or approach related emotional disturbances (103, 106). A diffusional kurtosis study has found disrupted superior and middle cerebellar peduncles in related to depression, with a particular relationship between disease duration and right SCP (107). The right posterior cerebellar white matter was also associated with treatment resistance in depression in a voxel-based DTI study (105). Functional brain studies have shown the involvement of cerebellar abnormality in depressed PD and also severe PD (108, 109). While connectivity of superior and middle cerebellar peduncles was significantly lower in related to comorbid RBD without depression in this study, reduced connectivity is seen only in right SCP, the main output route from the cerebellum to the left prefrontal cortex, in depressed PD patients with comorbid RBD and there is no such association with depressed PD without concomitant RBD. This may be a result of a small number of patients or may point to the specific patterns of comorbidity of depression and RBD in PD. Future studies with a larger number of patients are needed to investigate the generalizability of these results.

Another white matter structure with consistently differed connectivity in between-patients' subgroups comparison was fornix. Fornix, a limbic structure, is the main output tract of the hippocampus to diencephalon and basal forebrain. This structure is an important component of both episodic memory and emotional circuits (110–112). Fornix degeneration is proposed as a strong predictor of upcoming cognitive impairment, as it precedes hippocampal atrophy (113). Comorbid mild cognitive impairment and late-life depression, a possible representation of neurodegenerative disorders, has been related to reduced FA in fornix (114). This result has also been linked to treatment-refractory major depressive disorder (115). In another DTI studies, higher mean diffusivity (MD) in fornix has been revealed in PD patients (116) and with association with short-term nonverbal memory impairment in these patients (117). Experimental studies on animal models have demonstrated that so-called hypocretin neurons in perifornical region regulate sleep/wake cycle (118, 119) and neuronal loss may cause increased REM sleep portion (120, 121) and sleep disorders such as narcolepsy (122, 123). Interestingly, hypocretin neurotransmission system is shown to be affected in a post-mortem study of PD patients (124). Anatomical disruptions are also reported in iRBD (125) and the generation of excessive daytime sleepiness in PD patients (126). Our previous studies and the current study are the first to directly attribute fornix to comorbid depression and RBD in early PD. Left fornix disruption, resulted in disconnection of the left or verbal sphere of the hippocampus may contribute to memory deficits for verbal stimuli, in contrast to visuospatial input processed in the right side (127). Although executive dysfunction is considered as the hallmark cognitive deficit in PD, it has been cleared that verbal memory impairment has the greatest impact of all cognitive domains in PD (128–132). Poor performance on verbal memory tasks is also shown to be associated with sleep problems such as RBD in PD patients (133). Unsurprisingly, depressive disorders are accompanied by impairment in verbal memory as well (134).

There are some discrepancies in results from the current study and our two previous studies (Table 3). In order to control for the effect of the motor and other non-motor symptoms, we attempted to include patients with matched scores on other tests in the current study. This resulted in overlap in our patient selection from PPMI cohort. Using a new version of DSI studio may have also imposed more precise outcomes. Not using gold-standard diagnostic assessments for RBD and depression, polysomnography and clinically approved depression using DSM criteria, may be a source of error that should be kept in mind in interpreting our results. Despite GDS and RBDSQ scores, PD patients had worse olfaction function compared to HC and this would have contributed to the observed connectivity differences in the first part of the analysis. Longitudinal studies on larger subgroups of PD patients will address the accuracy of these results and better specify the role of each tract disruption in emergence of comorbid symptoms in heterogenous PD. In other words, tracts with differed connectivity in only within PD patients' comparison, such as cerebellar peduncles which most consistently were attributed to PD-RBD, and fornix in dPD-RBD may serve as markers useful for PD subtyping, which would be helpful in establishing better prognostic evaluation and more individualized treatment strategies. Needless to mention that these are preliminary findings that should be approved by future research.

## Conclusion

The results of this study support the entangled pathophysiology of depression and RBD which both predict poor outcomes regarding motor symptoms and cognitive decline in PD patients. As discussed above, specific commissural (CC), projection (CST, SCP, MCP) and long association fibers (cingulum, fornix, ILF, UF, IFOF) have been previously shown to serve as neural underpinnings of malignant subtypes of PD besides reputable associations with RBD and depression. So, disruption in these tracts may serve as an underlying pathology of REM sleep and mood dysregulations in early PD which also warn the emergence of debilitating motor and cognitive symptoms. A novel result of this study is the disruption of short association fibers (commonly known as U-fibers) in PD neurodegeneration that should be addressed in future research. A small number of patients in each group and not using gold standard tools to diagnose NMS in PPMI database should raise suspicion as multiple sources of error in this study. Shared neural substrates in RBD and depression in early PD is promising to discover high-risk individuals for future PD. Follow-up studies with larger number of patients will clear whether small regional diversities observed in between-patients-groups comparisons are linked to a specific pattern of RBD and depression in PD or may be justified by small sample sizes.

## Ethics statement

All procedures performed here, including human participants were in accordance with the ethical standards of the institutional research committee and with the 1964 Helsinki declaration and its later amendments or comparable ethical standards. Informed consent was obtained from all individual participants included in the study.

## Author contributions

FG and MA contributed to the conception and design of the study; AA-G, HS, FG, and MA contributed to data collection and analysis; FG, YR, MMZ and MA contributed to writing and revising the manuscript.

### Conflict of interest statement

The authors declare that the research was conducted in the absence of any commercial or financial relationships that could be construed as a potential conflict of interest.
